# Neural auditory encoding and performance in speech-in-noise perception: a pilot study

**DOI:** 10.1590/2317-1782/e20250032en

**Published:** 2025-12-01

**Authors:** Manoella Helena Lucera, Pamela Papile Lunardelo, Humberto de Oliveira Simões, Sthella Zanchetta

**Affiliations:** 1 Programa de Pós-Graduação em Clínica Médica, Departamento de Clínica Médica, Faculdade de Medicina de Ribeirão Preto, Universidade de São Paulo – USP - Ribeirão Preto (SP), Brasil.; 2 Departamento de Formação Específica em Fonoaudiologia, Universidade Federal Fluminense – UFF - Rio de Janeiro (RJ), Brasil.; 3 Departamento de Ciências da Saúde, Faculdade de Medicina de Ribeirão Preto, Universidade de São Paulo – USP - Ribeirão Preto (SP), Brasil.

**Keywords:** Hearing, Auditory Speech Perception, Frequency Following Response, Auditory Evoked Potential, Adults

## Abstract

**Purpose:**

To investigate and characterize the Frequency Following Response (FFR) and performance on the speech-in-noise test in adults who are native speakers of Brazilian Portuguese, as well as to assess the potential correlation between the two measures.

**Methods:**

A total of 28 individuals aged 18 to 29 years, with no diagnosis of hearing loss or history of conditions affecting hearing, participated in the study. Eligibility assessments included hearing sensitivity tests and mental status screening. The research assessments comprised the Portuguese Sentence List and FFR recordings.

**Results:**

The mean signal-to-noise ratio was -0.73 dB, ranging from -4.6 dB to 1.6 dB. In the FFR, all components were identified in 100% of participants, except for component C, which was present in 96.43%. A significant positive correlation was observed between the signal-to-noise ratio and the latencies of components A and C, while a significant negative correlation was found between the signal-to-noise ratio and the amplitudes of components A and D.

**Conclusion:**

FFR results determined characteristics of the present population, with component values similar to those reported in the Brazilian population. Better performance in speech-in-noise perception was correlated with shorter neural encoding time for the 'voice onset time' and greater neural recruitment for encoding the sound structure of the vowel.

## INTRODUCTION

Decoding of verbal stimuli involves multiple stages of auditory neural processing^(^1^)^. Speech is a complex stimulus that requires proper functioning of the auditory system for an accurate and sensitive analysis of its features. Among these features, the discrimination of variations in the temporal patterns of frequency and duration is particularly critical, especially in acoustically challenging environments^(^1^)^. This process occurs within the central auditory nervous system (CANS), beginning in the cochlear nuclei and extending to cortical areas^(^2^)^.

The Frequency-Following Response (FFR) is an auditory evoked potential that reflects neural activity synchronized with the encoding of speech stimuli^(^2^)^. This potential exhibits a stable morphology that is consistent with the acoustic input, with the synthetic syllable /da/, developed by Skoe and Kraus^(^3^)^, being the most commonly used. The components of the FFR reflect phase-locked neural activity during the encoding of the syllable and are divided into temporal and sustained portions, both of which are synchronized with the spectrotemporal characteristics of periodic sound^(^2^)^. The temporal portion, or “onset,” corresponds to the rapid acoustic features of the consonant and is represented by the V, A, and C components. The V–A complex reflects the onset of the consonant, whereas the C component represents the transition from the consonant to the vowel. The sustained portion, or “frequency following response,” corresponds to the periodic and harmonic structure of the vowel and is represented by the D, E, and F components. The O component reflects the final response, or “offset,” marking the decrease in neural firing synchrony^(^1,2^)^. The onset response is primarily generated in the lateral lemniscus and inferior colliculus. In contrast, sustained responses are generated in the cochlear nucleus, superior olivary complex, lateral lemniscus, and, predominantly in the inferior colliculus^(^4^)^.

FFR is a promising tool for investigating speech sound perception across diverse populations, both in the assessment and monitoring of therapeutic processes related to hearing and language^(^1,2^)^. To date, most studies on FFR have focused on conditions that negatively affect learning, such as dyslexia, academic difficulties, autism spectrum disorder, attention-deficit/hyperactivity disorder, specific language impairment, and central auditory processing disorder^(^1^)^. In the context of aging and hearing loss, FFR studies have shown that older adults and individuals with hearing loss exhibit reduced neural synchrony, particularly in noisy environments^(^1,4^)^.

FFR may reflect an individual’s linguistic experience; however, few studies have investigated correlations between this potential and performance on behavioral tests^(^5-7^)^. Bidelman and Momtaz^(^4^)^ demonstrated that more robust subcortical FFR responses are associated with better speech-in-noise perception in individuals with normal hearing, suggesting that subcortical structures preserve the neural periodicities essential for this skill. This finding is consistent with previous results in healthy adults^(^6^)^ and with a study that linked FFR to the perception of temporal aspects of auditory stimuli^(^7^)^, reinforcing the idea that neural encoding captured by FFR can offer valuable insights into how spectrotemporal features of speech are processed across different populations (e.g., due to linguistic experience, maturation, or aging). Another study demonstrated greater precision and stronger FFR activity in response to stimulus periodicity in speakers of tonal languages (e.g., Chinese) than in speakers of nontonal languages (e.g., English)^(^8^)^. This finding suggests that this potential is influenced by prior knowledge of language-specific acoustic patterns. Thus, the authors reported that FFR might reveal perceptual differences among native speakers of different languages.

Thus, although international studies have demonstrated a correlation between FFR and speech-in-noise perception, generalizing these findings to native speakers of languages not previously examined, such as Brazilian Portuguese, should be done with caution^(^5,6^)^. To date, we have not identified any studies that have investigated FFR in relation to speech-in-noise perception performance among healthy native speakers of Brazilian Portuguese. Given evidence suggesting that its components reflect linguistic experience, knowledge of a possible correlation between FFR results and this ability in native speakers of Brazilian Portuguese could support its application in individuals with speech perception difficulties.

The present study was based on the hypothesis that FFR components, by reflecting an individual's linguistic experience, are associated with better performance in speech-in-noise perception. Therefore, our objective was to investigate and characterize the FFR and speech-in-noise test performance of adult native speakers of Brazilian Portuguese, and to determine whether a correlation exists between the two measures.

## METHOD

This observational, cross-sectional study was conducted at the Ribeirão Preto Medical School of the University of São Paulo, following approval by the Ethics Committee (protocol no. 6,946,581) in 2024. All participants signed an informed consent form.

### Sample characteristics

A convenience sample of undergraduate and graduate students from the institution was used where the study was conducted. Participants were recruited through direct contact with researchers or announcements on social media platforms.

Individuals aged 18–29 years and 11 months were invited to participate based on the following inclusion criteria: native speakers of Brazilian Portuguese; no prior knowledge of hearing loss; no history of past or current use of ototoxic medications; no history of recurrent middle ear infections; and no history of traumatic brain injury, epileptic seizures, convulsions, migraine, or diabetes mellitus. On the day of the assessment, the exclusion criteria included the presence of hearing loss and/or a score below the expected threshold on the Mini-Mental State Examination. These criteria were established because of their potential to interfere with proposed assessment procedures^(^1,2^)^.

Initially, 34 individuals were selected; however, six were excluded: one because of altered auditory sensitivity, and five because their electrophysiological recordings were not analyzable due to a high number of artifacts later identified as a result of power line interference. The final sample consisted of 28 participants, of whom 16 (57.14%) were female and 12 (42.86%) were male, with a mean age of 21.96 years (SD = 2.56).

### Procedures

#### Eligibility phase

During the eligibility phase, procedures were conducted to ensure adequate auditory sensitivity, integrity of the tympano-ossicular system, and an appropriate mental status.

**Auditory Sensitivity** – Air conduction thresholds were assessed using a MADSEN Astera 2 audiometer with HDA 300 headphones at frequencies ranging from 0.25 to 8 kHz. Thresholds ≤ 15 dB HL were considered to be within normal limits^(^9^)^. Tympanometry was performed using an Otometrics ZODIAC 901 device with a 226 Hz probe tone. Compliance values between 0.3 and 1.7 ml, obtained within a pressure range of +50 to –150 daPa, were considered adequate^(^10^)^.

**Mental State** – The Mini-Mental State Examination (MMSE) was administered to minimize the possibility of cognitive impairment. Normal performance was determined based on criteria proposed in the Brazilian Portuguese-translated and validated versions of the instrument^(^11^)^.

#### Evaluation phase

**Speech-in-Noise Test** – The Portuguese Sentence Lists (Listas de Sentenças em Português - LSP) test was used^(^12^)^. The LSP provides a comparative analysis between the speech recognition threshold in noise and the intensity of the competing noise, resulting in the signal-to-noise ratio (SNR). Sentences were presented both with and without white noise (0.33 to 6.126 kHz). Initially, a training phase was conducted to obtain the sentence-recognition threshold for silence (List 1B). The speech recognition threshold in noise (List 2B) was determined, starting at an intensity of 65 dB(A) and an SNR of 0 dB, with intensity adjustments of 4 dB (+4 dB following errors and -4 dB following correct responses). After the first response change, intensity adjustments were made in 2 dB steps (+2 dB for errors and -2 dB for correct responses) until the end of the list (e.g., if the initial sentence was presented at 65 dB(A) and correctly identified, the intensity would decrease to 61 dB(A), continuing until an error occurred). From that point, the intensity varied in 2 dB steps, increasing after errors and decreasing after correct answers until completion of the list). The threshold was calculated as the average of the correct responses for the 10 sentences following the first response change, and the SNR value was obtained by subtracting this threshold from the noise intensity of 65 dB(A).

**Frequency-Following Response (FFR)** – The assessment was conducted using a Smart EP system (Intelligent Hearing Systems), a two-channel device with ER3A insert earphones. Skin was cleansed prior to electrode placement to remove epithelial debris and oil. Surface electrodes were positioned according to the International 10–20 System (1958)^(^13^)^, with electrode impedance maintained between 1 and 3 kΩ. The active (positive) electrode was placed at the vertex, the reference (negative) electrode on the right earlobe, and the ground electrode on the forehead. Stimulation parameters included the synthetic syllable /DA/ lasting 40 ms, presented monaurally at 80 dB HL at a rate of 10.9 stimuli per second with alternating polarity. Recording parameters comprised a bandpass filter of 100 to 3000 Hz, an amplification gain of 100 μV, and an analysis window from 0 to 64 ms.

Two sweeps of 1000 stimuli each were recorded. The resulting waveforms were summed, and the combined signal was analyzed by two evaluators, focusing on the latency and amplitude in the time domain. This analysis enabled the identification of the components V, A, C, D, E, F, and O.

### Statistical analysis

The inferential analysis was performed using SPSS Statistics version 22. Data distribution was assessed and found to be non-normal (Kolmogorov–Smirnov test). Correlations between the FFR and LSP results were analyzed using Spearman’s rank-order correlation (nonparametric). A significance level of 5% (p < 0.05) was used for the hypothesis testing.

## RESULTS

### Speech-in-Noise Test

The SNR results from the LSP test showed a mean value of -0.73 dB (standard deviation of 1.39 dB), with minimum and maximum values of -4.6 dB and 1.6 dB, respectively.

### Frequency-Following Response

**Figure 1:**
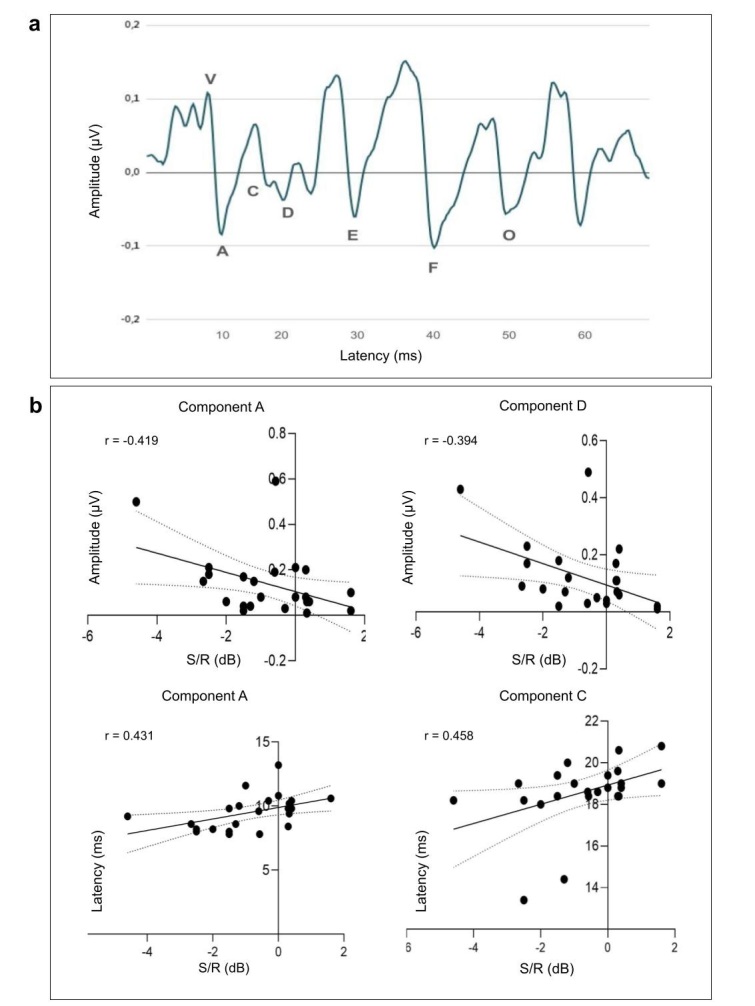
Grand average waveform and graphs of statistically significant correlations identified

Among the seven components of the FFR, all components were identified in 100% of the sample (n = 28), except for component C, which was present in 96.43% of the participants (27 of 28). Descriptive data for each component are presented in Table 1, and the grand average waveform derived from the dataset is shown in Figure 1a.

**Table 1:** Descriptive values from the Portuguese Sentence Lists test and Frequency-Following Response components

Portuguese Sentence Lists (*Listas de Sentenças em Português – LSP*)				
	Mean	Standard Deviation	Minimum	Maximum
**Sentence Recognition Threshold in Noise (dB)**	49.37	1.46	45.33	51.60
**Signal-to-Noise Ratio (dB)**	-0.73	1.39	-4.60	1.60
Frequency-Following Response				
	Latency (ms)		Amplitude (µV)	
Component (n)	Mean	Standard Deviation	Mean	Standard Deviation
**V (28)**	6.87	0.77	0.16	0.09
**A (28)**	9.59	1.29	0.12	0.13
**C (27)**	18.61	1.60	0.06	0.05
**D (28)**	22.96	1.00	0.11	0.11
**E (28)**	32.14	1.75	0.14	0.14
**F (28)**	40.73	1.30	0.12	0.14
**O (28)**	49.29	1.57	0.13	0.09

**Caption:** dB: decibel; ms: milliseconds; µV: microvolts

**Caption:** µV: microvolts; ms: milliseconds; S/N (dB): signal-to-noise ratio (decibels); r = correlation coefficient

### Speech-in-Noise Test vs. Frequency-Following Response

Spearman’s correlation analysis revealed statistically significant correlations (p < 0.05) between the SNR from the LSP test and components A, C, and D (Figure 1b). For the other components, correlations were not statistically significant (p > 0.05).

Regarding latency, significant positive correlations were observed between the SNR and components A (p = 0.021; r = 0.431) and C (p = 0.014; r = 0.458), indicating that shorter neural response times are associated with better speech-in-noise perception performance, that is, with a more negative SNR (dB) value. A significant negative correlation was also identified between the SNR and the amplitudes of components A (p = 0.026; r = -0.419) and D (p = 0.042; r = -0.394), suggesting that greater amplitude was associated with better speech-in-noise perception performance, corresponding to a more negative SNR (dB) value (Figure 1b).

## DISCUSSION

### Speech-in-Noise Test

In the LSP test, the observed SNR was higher than that reported for adults of the same age range in a previous study^(^14^)^. Because no normative reference values have been established for this test, it was not possible to determine whether the results indicated typical or impaired performance in either study. However, it is worth noting that a negative SNR is frequently associated with good performance in speech perception^(^14^)^.

### Frequency-Following Response

The latency and amplitude values observed in the FFR were consistent with those reported in a previous study involving young, normal-hearing female participants^(^15^)^, indicating similar patterns of neural responses to auditory stimuli. The absence of the C wave in one participant is supported by literature, which reports its occasional absence, even in reliable recordings obtained from both young and older adults^(^16^)^.

### Speech-in-Noise Test vs. Frequency-Following Response

These results indicate that a more negative SNR is associated with shorter neural response times for components A and C. This suggests that better speech-in-noise perception is associated with faster neural encoding of voice onset time (VOT), that is, the detection of rapid temporal changes characteristic of consonants. In the case of the plosive consonant /d/, the VOT was particularly short (16 ms), characterizing a rapid transition between the consonant and vowel. This phenomenon involves a quick formant transition, resulting in a “glide” that facilitates perception and is represented by component C. Because perceptual processing of this stimulus requires rapid temporal analysis^(^1,2^)^, its association with latency is understandable.

Better speech perception was also associated with greater neural recruitment, as reflected in waves A and D. This demonstrates that higher amplitudes in these components are related both to rapid consonant analysis and to the encoding of the vowel’s periodic and harmonic structure. Components D, E, and F reflect synchronization with the fundamental frequency (F_0_), and the interval between D and E provides information about the phase locking at the frequency of the first formant (F1)^(^2^)^. In this study, the phenomenon of phase locking to F1 indicated that more synchronized neural encoding of the spectrotemporal properties of the sound wave was associated with better speech-in-noise perception. This finding aligns with the hypothesis that neural oscillations synchronized with F1 are essential for speech recognition (e.g., vowel identification and timbre perception).

These results are consistent with findings from previous English-language studies that hypothesized a possible association between behavioral performance in speech perception and electrophysiological findings from the FFR, given that both rely on subcortical neural synchrony. In line with our findings, a study using electroencephalographic recordings of the FFR demonstrated that faster and more robust encoding is associated with better speech-in-noise perception^(^4^)^. The same study confirmed that, although multiple auditory neural generators contribute to the formation of the FFR, the inferior colliculus is the primary source^(^4^)^. These findings highlight the FFR as a potential tool for studies investigating the physiological mechanisms underlying the subcortical neural encoding of speech.

These findings revealed patterns of neural encoding in native speakers of Brazilian Portuguese and their relationship to speech perception under adverse listening conditions. The relevance of these results lies in the crucial role of native language acquisition in shaping the neural architecture responsible for detecting auditory and linguistic patterns^(^4,5^)^, which may have predictive implications for future abilities. It is important to emphasize that this neural synchrony reflects specific characteristics of Brazilian Portuguese, making it inappropriate to directly extrapolate findings from studies conducted in other languages. This evidence contributes to our understanding of the neural mechanisms underlying speech-in-noise perception and provides a foundation for investigating individual variability in auditory-linguistic skills.

### Study limitations

A limitation of this study is its small sample size; however, it was sufficient as an initial investigation and provides direction for future research. Applying the present protocol to populations with different linguistic experiences—such as bilingual individuals, those with musical training, or individuals with hearing loss—represents a promising direction for further studies.

## CONCLUSION

The FFR results identified characteristics specific to the study population, with component values similar to those previously reported for the Brazilian population. Better performance in speech-in-noise perception was correlated with shorter neural encoding of the voice onset time and greater neural recruitment for encoding the vowel’s sound structure.
