# Supplementary material for: Neural auditory encoding and performance in speech-in-noise perception: a pilot study
Source: Codas. 2025 Dec 1;37(6):e20250032. doi: 10.1590/2317-1782/e20250032en (PMC12704604; doi:10.1590/2317-1782/e20250032en)
Supplement: Translation

# Translation: Codificação neural auditiva e o desempenho na percepção de fala no ruído: um estudo piloto

**DOI:** 10.1590/2317-1782/e20250032pt

**Authors:** Manoella Helena Lucera, Pamela Papile Lunardelo, Humberto de Oliveira Simões, Sthella Zanchetta

**Affiliations:** 1 Programa de Pós-Graduação em Clínica Médica, Departamento de Clínica Médica, Faculdade de Medicina de Ribeirão Preto, Universidade de São Paulo – USP - Ribeirão Preto (SP), Brasil.; 2 Departamento de Formação Específica em Fonoaudiologia, Universidade Federal Fluminense – UFF - Rio de Janeiro (RJ), Brasil.; 3 Departamento de Ciências da Saúde, Faculdade de Medicina de Ribeirão Preto, Universidade de São Paulo – USP - Ribeirão Preto (SP), Brasil.

**Keywords:** Audição, Percepção de Fala, Frequency Following Response, Potencial Evocado Auditivo, Adultos

## Abstract

**Objetivo:**

Investigar e caracterizar o FFR e o desempenho no teste de fala no ruído em adultos falantes do português brasileiro, como língua materna, bem como, verificar se existe correlação entre os dois testes.

**Método:**

Participaram 28 indivíduos, entre 18 e 29 anos, sem perda auditiva. Foram realizadas avaliações de elegibilidade, como a determinação da sensibilidade auditiva e a triagem do estado mental. As avaliações de pesquisa incluíram a Lista de Sentenças em Português e o FFR.

**Resultados:**

Os resultados mostraram que a relação sinal-ruído média foi de -0,73 dB, com valores variando de -4,6 dB a 1,6 dB. No FFR, todas as componentes foram identificadas em 100% dos participantes, exceto a componente C, presente em 96,43%. Foi observada uma correlação significativa e positiva entre a relação sinal-ruído e as latências das componentes A e C, e uma correlação significativa e negativa entre a relação sinal-ruído e as amplitudes das componentes A e D.

**Conclusão:**

Os resultados do FFR determinaram características da presente população, com valores das componentes semelhantes aos relatados na população brasileira. O melhor desempenho na percepção de fala no ruído foi correlacionado a um menor tempo para codificação neural do “voice onset time” e maior recrutamento neural para codificação da estrutura sonora da vogal.

## INTRODUÇÃO

A decodificação dos estímulos verbais envolve vários estágios do processamento neural auditivo^(1)^. A fala é um estímulo complexo que demanda o funcionamento adequado do sistema auditivo para a sensível e precisa análise de suas características. Entre essas características destacam-se, particularmente em ambientes acústicos desfavoráveis, a discriminação de variações nos padrões temporais de frequência e duração^(1)^. Esse processamento ocorre no sistema nervoso auditivo central (SNAC), que tem seu início nos núcleos cocleares se estendendo às áreas corticais^(2)^.

O *Frequency Following Response* (FFR) é um potencial evocado auditivo que reflete a atividade neural sincronizada à codificação de estímulos de fala^(2)^. O potencial apresenta morfologia estável e consistente com o *input acústico,* sendo a sílaba sintética /da/, produzida por Skoe e Kraus^(3)^, a mais utilizada. As componentes do FFR refletem a atividade neural *phase-locked* durante a codificação da sílaba, dividindo-se em porção temporal e contínua, que sincronizam com as características espectro-temporais deste som periódico^(2)^. A porção temporal ou “*onset*” corresponde às características acústicas rápidas da consoante, representada pelas componentes V, A, C. O complexo V-A responde ao início da consoante, enquanto a componente C à transição entre a consoante e a vogal. A porção contínua ou “*frequency following response*” é uma resposta à estrutura sonora periódica e harmônica da vogal, representada pelas componentes D, E e F. A componente O representa a resposta final ou “*offset*”, marcando a redução da sincronia de disparo neural^(1,2)^. A resposta da porção temporal é produzida no lemnisco lateral e colículo inferior, enquanto as repostas da porção contínua são geradas pelo núcleo coclear, complexo olivar superior, lemnisco lateral e, principalmente, pelo colículo inferior^(4)^.

O FFR é uma ferramenta promissora para a investigação da percepção dos sons da fala em diferentes populações, tanto na avaliação quanto no monitoramento de processos terapêuticos relacionados à audição e à linguagem^(1,2)^. Até o momento, o maior interesse pelo FFR tem se concentrado em condições que afetam negativamente a aprendizagem, como dislexia, dificuldades escolares, transtorno do espectro autista, transtorno do déficit de atenção e hiperatividade, transtorno específico de linguagem e transtorno do processamento auditivo central^(1)^. No contexto do envelhecimento e da perda auditiva, o FFR tem evidenciado que adultos mais velhos e/ou com perda auditiva apresentam uma sincronia neural reduzida, especialmente em ambientes com ruído^(1,4)^.

O FFR pode refletir a experiência linguística do sujeito, entretanto poucos estudos dedicaram-se na busca por correlações entre esse potencial com o desempenho em testes comportamentais^(5-7)^. Bidelman e Momtaz^(4)^ demonstraram que respostas subcorticais mais robustas do FFR correlacionaram-se com o melhor desempenho da percepção de fala no ruído em normo-ouvintes, sugerindo que essas estruturas mantêm periodicidades neurais essenciais para esta habilidade. Este resultado está de acordo com achados prévios em adultos saudáveis^(6)^ e com um estudo que correlacionou o FFR à percepção de aspectos temporais^(7)^, reforçando que a codificação neural capturada pelo FFR pode oferecer *insights* valiosos sobre como as características espectro-temporais da fala são processadas em diferentes populações (e.g. experiência linguística, maturação, envelhecimento). Outro estudo demonstrou maior precisão e atividade neural mais intensa do FFR em resposta à periodicidade do estímulo em falantes de línguas tonais (como o chinês) em comparação com falantes de línguas não-tonais (como o inglês)^(8)^. O resultado sugere que o potencial é influenciado pelo conhecimento prévio dos padrões acústicos específicos da língua, sendo assim, os autores relataram que ele pode evidenciar diferenças perceptuais entre falantes nativos de diferentes línguas.

Desta forma, ainda que estudos internacionais tenham evidenciado uma correlação entre o FFR e a percepção de fala no ruído, a generalização destes resultados para falantes nativos de diferentes línguas daquelas dos estudos acima, como o português-brasileiro, deve ser feita com cautela^(5,6)^. Até o presente momento, não identificamos estudos que tenham investigado o FFR em relação ao desempenho da percepção de fala no ruído, em falantes nativos do português-brasileiro hígidos. Partindo do pressuposto que há evidências que suas componentes refletem experiência linguística, o conhecimento de uma possível correlação entre seus resultados com essa habilidade em falantes nativos do português-brasileiro pode contribuir para seu uso em sujeitos com queixa de dificuldade de percepção de fala.

O presente estudo foi desenvolvido com a hipótese de que as componentes do FFR, por refletirem a experiência linguística do sujeito, estarão relacionadas ao melhor desempenho da fala na presença de ruído. Sendo assim, nosso objetivo foi investigar e caracterizar o FFR e o desempenho no teste de fala no ruído em adultos falantes do português brasileiro, como língua materna, bem como, verificar se existe correlação entre os dois testes.

## MÉTODO

Estudo observacional e transversal realizado na Faculdade de Medicina de Ribeirão Preto da Universidade de São Paulo, após aprovação no Comitê de Ética (nº 6.946.581) no ano de 2024. Todos os sujeitos assinaram o termo de consentimento livre e esclarecido.

### Casuística

A amostra foi composta por conveniência, todos os sujeitos eram estudantes de graduação ou pós-graduação da instituição onde o estudo foi conduzido. Os sujeitos foram recrutados a partir do contato direto das pesquisadoras ou da divulgação em mídias sociais.

Foram convidados sujeitos entre 18 e 29 anos e 11 meses com os seguintes critérios: português-brasileiro como língua materna; sem conhecimento prévio de possuir perda auditiva; não fazer uso de medicamentos ototóxicos – prévios ou atuais; não apresentar histórico de infecção recorrente de orelha média; não apresentar antecedentes de traumatismo crânio-encefálico, crises de epilepsia e convulsão, migrânea ou diabetes mellitus. No dia da avaliação foram considerados critérios de exclusão a presença de perdas auditivas e/ou o escore inferior ao esperado no Mini Exame do Estado Mental. Esses critérios foram estabelecidos por representarem potenciais fatores de interferência na avaliação proposta^(1,2)^.

Inicialmente foram selecionados 34 sujeitos, entretanto seis deles foram excluídos, um por alteração da sensibilidade auditiva e cinco por seus registros eletrofisiológicos não serem passíveis de análise, devido ao grande número de artefatos, posteriormente identificados como decorrentes de interferências na rede de energia. A amostra final foi composta por 28 sujeitos, dos quais 16 (57,14%) eram do sexo feminino e 12 (42,86%) do sexo masculino, com média de idade de 21,96 anos (desvio padrão = 2,56).

### Procedimentos

#### Etapa de elegibilidade

Na etapa de elegibilidade foram aplicados procedimentos que asseguraram a adequada sensibilidade auditiva, integridade do funcionamento tímpano-ossicular e estado de consciência mental.

**Sensibilidade auditiva** – A pesquisa dos limiares aéreos foi realizada com o audiômetro MADSEN Astera 2, fone HDA 300, nas frequências de 0,25 a 8 kHz, considerando normais aqueles ≤ 15 dB NA^(9)^. A timpanometria foi realizada com o equipamento da marca Otometrics – ZODIAC 901, com sonda de 226 Hz, considerando como adequados os valores de complacência entre 0,3 a 1,7 ml obtidos entre +50 e -150 daPA^(10)^.

**Estado de consciência mental** – Foi aplicado o Mini Exame do Estado Mental para minimizar a possibilidade da presença de alteração cognitiva. Para normalidade, considerou-se o proposto na versão traduzida e validada para o português-brasileiro^(11)^.

#### Etapa de avaliação

**Teste de Fala no Ruído** – Foi utilizado o teste Listas de Sentenças em Português (LSP)^(12)^. O LSP oferece uma análise comparativa entre o limiar de reconhecimento de sentenças no ruído e a intensidade do ruído competitivo, resultando na relação S/R. As sentenças foram apresentadas com e sem ruído branco (0,33 a 6,126 kHz). Inicialmente, foi realizada a etapa de treino para obter o limiar de sentenças no silêncio (lista 1B). O limiar de reconhecimento de sentenças no ruído (lista 2B) foi determinado com intensidade inicial de 65 dB(A) e relação S/R de 0 dB, variando-se a intensidade em 4 dB (+4 dB para erros e -4 dB para acertos). Após a primeira mudança de resposta, a intensidade foi ajustada em 2 dB (+2 dB para erros e -2 dB para acertos), até o término da lista (e.g. sentença inicial apresentada a 65 dB(A), em caso de acerto, a intensidade reduziria para 61 dB(A) e assim por diante, até um erro. A partir disso, a intensidade variaria em passos de 2 dB, aumentando para erros e diminuindo para acertos, até o fim da lista). O limiar foi calculado pela média dos acertos nas 10 sentenças após a primeira mudança de resposta, e o valor S/R foi a diferença entre esse limiar e o ruído de 65 dB(A).

**Frequency Following Response** – Realizado com o equipamento Smart EP - *Intelligent Hearing System*, de dois canais, com fone de inserção ER3A. Foi realizada a limpeza de pele para a remoção de resíduos de descamação epitelial e de oleosidade, prévia à fixação dos eletrodos de superfície, dispostos de acordo com a norma internacional 10-20 (1958)^(13)^ e com nível de impedância mantido entre 1-3 Kohms. O eletrodo ativo-positivo foi fixado no vértex, o eletrodo referência-negativo no lóbulo direito e o eletrodo Terra na fronte. Os parâmetros de estimulação foram a sílaba sintética /DA/ de 40 ms, apresentação monaural a 80 dB NA, taxa de 10,9 estímulos por segundo e polaridade alternada. Para a captação foi utilizado filtro de 100 a 3000 Hz, ganho de 100 μV e janela de análise de 0 a 64 ms.

Foram realizadas duas varreduras, cada uma composta por 1000 estímulos. Os traçados resultantes foram somados, e o sinal combinado foi analisado por dois avaliadores, considerando as variáveis de latência e amplitude no domínio do tempo. A análise resultou na identificação das componentes V, A, C, D, E, F e O.

### Análise estatística

A análise inferencial foi realizada utilizando o SPSS Statistics V22. A análise dos dados não apresentou distribuição normal (teste de Kolmogorov-Smirnov). A correlação dos resultados do FFR e o LSP foi realizada com o teste de Spearman (não paramétrico). Para os testes de hipótese, adotou-se o nível de significância de 5% (p<0,05).

## RESULTADOS

### Teste de Fala no Ruído

Os resultados da relação S/R do teste LSP apresentaram valores médios de -0,73 dB (desvio padrão de 1,39 dB), com valores mínimo e máximo de -4,6 dB e 1,6 dB, respectivamente.

### Frequency Following Response

Das sete componentes do FFR, exceto a componente C, presente em 96,43% dos sujeitos (27/28), todas as outras foram identificadas em 100% da amostra (n=28). Os dados descritivos das componentes estão apresentados na Tabela 1 e a onda média derivada do conjunto de dados na Figura 1a.

**Tabela 1:** Valores descritivos do teste Listas de Sentenças em Português e das componentes do *Frequency Following Response*

Listas de Sentenças em Português				
	Média	Desvio padrão	Mínimo	Máximo
**Limiar de Reconhecimento de Sentença no Ruído (dB)**	49,37	1,46	45,33	51,60
**Relação Sinal/Ruído (dB)**	-0,73	1,39	-4,60	1,60
*Frequency Following Response*				
	Latência (ms)		Amplitude (µV)	
Componente (n)	Média	Desvio padrão	Média	Desvio padrão
**V (28)**	6,87	0,77	0,16	0,09
**A (28)**	9,59	1,29	0,12	0,13
**C (27)**	18,61	1,60	0,06	0,05
**D (28)**	22,96	1,00	0,11	0,11
**E (28)**	32,14	1,75	0,14	0,14
**F (28)**	40,73	1,30	0,12	0,14
**O (28)**	49,29	1,57	0,13	0,09

**Legenda:** dB: decibel, ms: milissegundos, µV: microvolts

**Figura 1:** Onda média derivada do conjunto de dados (*grand average*) e gráficos das correlações estatisticamente significativas identificadas

**Legenda:** µV: microvolts, ms: milissegundos, S/R (dB): relação sinal-ruído (decibel), r= coeficiente de correlação

### Teste de Fala no Ruído vs. Frequency Following Response

O resultado do teste de Spearman demonstrou uma correlação estatisticamente significante (p < 0,05) entre a S/R do teste LSP com as componentes A, C e D (Figura 1b), para as outras componentes a correlação não foi significativa (p>0,05).

Em relação à latência, observou-se uma correlação significativa e positiva entre a relação S/R e as componentes A (*p* = 0,021; r = 0,431) e C (*p* = 0,014; r = 0,458), o que demonstra que os tempos de resposta neural mais curtos estão correlacionados a um melhor desempenho na percepção de fala no ruído, ou seja, a uma relação S/R (dB) mais negativa. Também foi identificada uma correlação significativa e negativa entre a relação S/R e as amplitudes das componentes A (*p* = 0,026; r = -0,419) e D (*p* = 0,042; r = -0,394), sugerindo que uma maior amplitude está correlacionada a um melhor desempenho da percepção de fala no ruído, ou seja, a uma relação S/R (dB) mais negativa (Figura 1b).

## DISCUSSÃO

### Teste de Fala no Ruído

No teste LSP a relação S/R observada foi superior à relatada em adultos de mesma faixa etária em um estudo prévio^(14)^. Como não há valores de referência de normalidade para o teste, não é possível determinar se os resultados indicam desempenho adequado ou alterado em nenhum dos dois trabalhos. Contudo, é pertinente ressaltar que relações S/R negativas são frequentemente associadas a um bom desempenho na percepção de fala^(14)^.

### Frequency Following Response

Os valores de latência e amplitude do FFR observados foram consistentes com uma pesquisa anterior com jovens normo-ouvintes do sexo feminino^(15)^, o que demonstra padrões semelhantes de resposta neural ao estímulo sonoro. A ausência da onda C em um dos sujeitos é corroborada com a literatura, que aponta para sua ausência ocasional, mesmo em registros confiáveis obtidos em jovens adultos e adultos mais velhos^(16)^.

### Teste de Fala no Ruído *vs.* Frequency Following Response

Os resultados indicam que, quanto mais negativa for a relação S/R, menor é o tempo de resposta neural para os componentes A e C. Isso sugere que um melhor desempenho na percepção de fala no ruído está associado a uma codificação neural mais rápida do *voice onset time* (VOT), ou seja, da detecção de mudanças temporais rápidas e intrínsecas das consoantes. No caso da consoante oclusiva /d/, o VOT é particularmente curto (16 ms), caracterizando uma transição rápida entre consoante e vogal. Esse fenômeno envolve uma mudança rápida entre formantes, resultando em um '*glide*' que facilita a percepção e é representado pela componente C. Como esses processos perceptivos do estímulo requerem análises temporais rápidas^(1,2)^, é compreensível que estejam associados à variável latência.

A melhor percepção de fala também foi associada a um maior recrutamento neuronal, refletido nas ondas A e D. Isto demonstra que uma maior amplitude dessas componentes está relacionada tanto à análise rápida da consoante quanto à codificação da estrutura sonora periódica e harmônica da vogal. As componentes D, E e F evidenciam a sincronização com a frequência fundamental (F0); além disso, o intervalo entre D e E fornece informações sobre o bloqueio de fase — ou *phase locking* — na frequência do primeiro formante (F1)^(2)^. Neste estudo, o fenômeno de bloqueio de fase para F1 indica que uma codificação neural mais sincronizada com as propriedades espectro-temporais da onda sonora está associada a um melhor desempenho na percepção de fala no ruído, convergindo com a hipótese de que oscilações neurais sincronizadas a F1 são essenciais para o reconhecimento de fala (e.g. identificação de vogais e na percepção do timbre).

Os resultados são concordantes com estudos anteriores em inglês que apontaram como hipótese uma possível associação entre o desempenho comportamental da percepção de fala e achados eletrofisiológicos do FFR, uma vez que ambos precisam da sincronia neural subcortical. Em consonância com nossos achados, um estudo que registrou o FFR por eletroencefalograma, demonstrou que a codificação mais rápida e robusta estava associada à melhor percepção de fala no ruído^(4)^. Este mesmo estudo confirmou que embora múltiplos geradores neurais auditivos contribuam para a formação do FFR, o colículo inferior é o principal contribuinte^(4)^. Esses achados ressaltam o FFR como uma ferramenta em potencial para estudos que buscam informações fisiológicas quanto à codificação neural da fala subcortical.

Estes achados revelam os padrões de codificação neural em falantes nativos do português brasileiro e sua relação com a percepção em condições adversas de escuta. A relevância desses resultados fundamenta-se no papel crucial da aprendizagem da língua materna na moldagem da arquitetura neural responsável pela detecção de padrões auditivos e linguísticos^(4,5)^, com implicações preditivas para habilidades futuras. Destaca-se que este sincronismo neural apresenta especificidades do português brasileiro, impossibilitando extrapolações diretas de estudos em outras línguas. Tais evidências contribuem para a compreensão dos mecanismos neurais subjacentes à percepção de fala no ruído, fornecendo subsídios para investigações sobre variabilidade individual em habilidades auditivo-linguísticas.

### Limitações do estudo

Como limitação, este estudo foi conduzido com tamanho amostral reduzido, entretanto, suficiente para um estudo inicial cujo resultado direciona para pesquisas futuras. O uso do presente protocolo em populações com diferentes experiências linguísticas — como bilíngues, indivíduos com experiência musical ou com perdas auditivas — constitui uma das possibilidades para investigações futuras.

## CONCLUSÃO

Os resultados do FFR determinaram características da presente população, com valores das componentes semelhantes aos relatados na população brasileira. O melhor desempenho na percepção de fala no ruído foi correlacionado a um menor tempo para codificação neural do “*voice onset time*” e maior recrutamento neural para codificação da estrutura sonora da vogal.
